# Ototoxicity associated with extended dalbavancin treatment for a shoulder prosthetic joint infection

**DOI:** 10.1186/s12879-023-08709-8

**Published:** 2023-10-19

**Authors:** Anna Lange, Ulrica Thunberg, Bo Söderquist

**Affiliations:** 1 Department of Infectious Diseases, Faculty of Medicine and Health, Örebro University, Örebro, Sweden; 2 Department of Otorhinolaryngology, Faculty of Medicine and Health, Örebro University, Örebro, Sweden; 3 School of Medical Sciences, Faculty of Medicine and Health, Örebro University, Örebro, Sweden

**Keywords:** Dalbavancin, Prosthetic joint infection, Ototoxicity

## Abstract

**Background:**

Dalbavancin is a lipoglycopeptide antibiotic approved for treatment of skin and soft tissue infections, administered as a single or two-dose treatment. The extended half-life, good penetration into bone and synovial fluid, and bactericidal activity against gram-positive bacteria, including those in biofilm, make dalbavancin an appealing choice for treatment of bone and joint infections in outpatient settings. However, we present a rare case of ototoxicity associated with off-label extended dalbavancin treatment of a prosthetic joint infection.

**Case presentation:**

A 55-year-old man with a prosthetic joint infection of the shoulder underwent off-label extended dalbavancin treatment, receiving a cumulative dose of 2500 mg. The patient experienced a gradual onset of hearing loss following the first dose, leading to a diagnosis of bilateral sensorineural hearing loss that persisted 1 year after dalbavancin was discontinued.

**Conclusions:**

This case report highlights the importance of exercising caution when administering dalbavancin beyond approved dosing guidelines, and emphasizes the need for vigilance regarding the potential for ototoxicity.

## Background

Arthroplasty surgery of the shoulder is a procedure that significantly improves quality of life for many patients. However, serious complications such as deep infections occur in 1–2% of cases [1, 2]. A prosthetic joint infection (PJI) often causes long-term suffering for the patient, as it generally requires repeated surgery and long-term antibiotic treatment and may lead to significant disability as a final outcome [3]. There are also substantial costs to the healthcare system [2], due to repeated surgery and prolonged and recurrent hospital stays [4].

The most common bacteria involved in the majority of PJIs are staphylococci, both *Staphylococcus aureus* and coagulase-negative staphylococci (CoNS), predominantly *Staphylococcus epidermidis*. However, one specific pathogen predominantly associated with shoulder PJIs is *Cutibacterium acnes* (previously known as *Propionibacterium acnes*) [5, 6]. Treatment of PJIs is often challenging, since most causative microorganisms produce biofilm, and in addition, many bacteria, such as CoNS, often display multi-drug resistance [7], making it difficult to find an effective oral treatment regimen.

Dalbavancin is a lipoglycopeptide with broad anti-microbial spectrum against gram-positive bacteria and a potential effect against microorganisms in biofilm [8, 9]. It is approved for treatment of bacterial skin and soft tissue infections (SSTI) caused by gram-positive microorganisms, and is recommended as a two-dose (1000 mg and 500 mg with a 1-week interval) or single-dose regimen (1500 mg) [10]. Its pharmacokinetic features, with an exceptional long half-life of approximately 300 h, enable an administration schedule that could also be an option for long-term treatment of bone and joint infections. Dalbavancin demonstrates good penetration into bone and synovial fluid, and has been shown to be efficacious and safe for the treatment of osteomyelitis in adults [11]. A number of clinical series with real-world experience with dalbavancin, have been published, encompassing cases with PJI [12]. A multicentre case series (*n* = 101) explored the off-label utilization of dalbavancin in various conditions such as PJI, osteomyelitis, endocarditis and catheter-related bloodstream infection. This series reported loading doses between 1000 and 1500 mg and a wide range of subsequent dosing regimens, spanning from a single 1500 mg dose after one week to as many as 32 doses at 500 mg administered weekly or 1000 mg administered bi-weekly [13]. The optimal dosage regimen and duration of treatment for bone and joint infections, including PJIs, remains to be established.

The safety and tolerability of dalbavancin have been reported to be very good [14–16], and this has been confirmed by several real-life studies [17–19]. However, there may be a concern regarding the ototoxic risk of glycopeptides, such as vancomycin [20, 21].

We here present a case of a patient with a shoulder PJI treated with debridement, antibiotics, and implant retention (DAIR), who was intended to undergo long-term dalbavancin therapy but experienced serious ototoxic adverse effect during treatment.

## Case presentation

The patient was a 55-year-old white man, a previous smoker, with a history of hypertension (ongoing medication enalapril 10 mg), pituitary adenoma, and tinnitus, but no hearing loss. He presented a complicated history of a PJI that began after a total left shoulder arthroplasty in October 2019 due to osteoarthritis as follows. The first reoperation was performed 2 months after primary surgery, due to subscapular tendon rupture. Debridement, irrigation, and exchange of modular components was performed. Cultures showed growth of *C. acnes* in 2/5 intraoperative tissue samples, susceptible to benzylpenicillin and clindamycin, and treatment with oral amoxicillin 1 g t.i.d. for 3 months was initiated. Due to persisting shoulder pain and impaired mobility, the patient was subjected to a two-stage revision surgery 7 months after the second operation. In first session of the two-stage procedure, the prosthetic devices were removed, and a spacer coated with gentamicin-containing bone-cement, bone cement containing both gentamicin and clindamycin, and a gentamicin-impregnated collagen sponge were all applied. Intraoperative cultures still showed growth of *C. acnes* with the same antibiotic susceptibility pattern in 4/7 samples, and the patient was prescribed oral clindamycin 300 mg t.i.d. for 3 months. After an antibiotic-free interval of four weeks, implantation of a reverse total shoulder prosthesis in the second session of the two-stage procedure was performed in October 2020. During this surgery bone fixation cement containing both gentamicin and vancomycin was used. However, intraoperative cultures again displayed growth of *C. acnes*, in 1/6 samples, and another 3-month oral course of amoxicillin was initiated. Due to an untenable pain situation, a fifth operation according to the DAIR (Debridement, Antibiotics, and Implant Retention) procedure was performed in September 2021. This time, intraoperative cultures displayed growth of both *C. acnes* (1/6 samples) and two strains of *S. epidermidis* (both 2/6). Neither of the *S. epidermidis* strains was susceptible to ciprofloxacin or clindamycin, but both strains, as well as the *C. acnes,* were susceptible to vancomycin. To enable outpatient treatment, intravenous dalbavancin (1000 mg initial dose followed by three weekly doses of 500 mg) was started. No therapeutic drug monitoring was performed since this was not available.

**Fig. 1:**
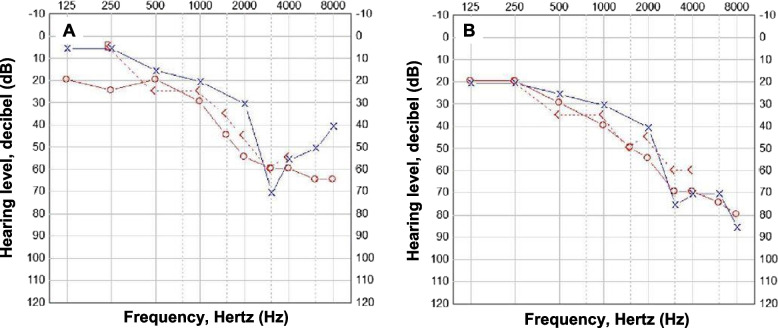
Audiograms of a patient with a prosthetic shoulder infection reporting hearing loss (**a**) after four doses of dalbavancin, and (**b**) at the one-year follow-up visit. Circles represent the right ear and crosses represent the left ear. X-axis: sound frequencies (Hz); y-axis: volume (dB). Pure Tone average (PTA^4^) is the mean of frequencies 0.5, 1, 2, and 4 kHz. PTA^4^ < 20 dB is regarded as normal hearing, PTA^4^ 20 < 35 dB as mild hearing loss and PTA^4^ 35 < 49 dB as moderate hearing loss

At a follow-up visit four weeks later, after four doses of dalvavancin at a cumulative dose of 2500 mg, the patient reported that since the first dose of dalbavancin he had noticed a gradual decrease in his hearing ability. He denied former hearing problems, but this could not be objectively verified due to lack of previously performed audiometry. Audiometry showed bilateral sensorineural hearing loss with an air-conducted pure tone average (PTA) of 49 dB in the right ear and 41 dB in the left ear (Fig. 1a). PTA was calculated as the average for the frequencies 0.5, 1, 2, and 4 kHz. According to classification by The World Health Organization (WHO) in 2008 for hearing impairment, a PTA^4^ < 20 dB is regarded as normal hearing, PTA^4^ 20 < 35 dB as mild hearing loss and PTA^4^ 35 < 49 dB as moderate hearing loss, at an eight-point scale [22]. In the higher frequency spectrum high-pitch voices and consonants such as “t”, “h”, “s”, and “f” will be found, which can be difficult to discriminate for individuals with reduced treble hearing. Dalbavancin was immediately discontinued, and the patient’s concomitant medications (amlodipine, testosterone, bromocriptine, and oxycodone) were reviewed for interactions and ototoxic potential, none of which were noted (Electronic medicines compendium in Sweden (www.fass.se). Another 2 months of antibiotics (amoxicillin 1 g t.i.d.) was prescribed for the PJI. Follow-up audiograms showed unresolved hearing loss, with an air-conducted PTA of 41 dB in the right ear and 30 dB in the left ear (Fig. 1b), and the patient was prescribed hearing aids. Two months after the discontinuation of antibiotics, the patient’s shoulder pain had diminished relative to the pain situation before the fifth operation.

## Discussion

This is, to our knowledge, the first report of suspected ototoxicity manifested by hearing loss associated with dalbavancin treatment. Our patient had a history of tinnitus, and there was no previous audiogram for comparison. It is possible that there was a pre-existing hearing impairment that was not previously clearly noticed by the patient and that was aggravated by dalbavancin-induced ototoxicity. The concomitant medication comprised amlodipine, testosterone, bromocriptine, and oxycodone, none of which are known to display an ototoxic potential. In addition, during the surgical procedures in 2020, the patient received antibiotic-loaded cement containing gentamicin, and a local application of gentamicin in collagen was administered. However, the systemic concentrations resulting from locally administered gentamicin are negligible [23], indicating that gentamicin is unlikely to have contributed significantly to the observed ototoxicity in our patient.

The safety of dalbavancin has been evaluated in several reviews [14, 15, 24]. Phase II and phase III clinical trials of acute bacterial skin and skin structure infections have reported low frequencies of adverse events, mainly mild [14, 15]. In addition, retrospective observational cohort studies show only rare occurrences of adverse events following off-label use or extended use of dalbavancin with varying dosing regimens and therapy duration, beyond the one- or two-dose regimen recommended for SSTI, especially for bone and joint infections [25] and infective endocarditis [26]. Morata et al. [24] performed a multicentre study of patients with osteoarticular infections receiving prolonged therapy. Out of 64 patients, seven reported adverse events such as gastrointestinal problems, rash, phlebitis, asthenia, and one case of increased serum creatinine. The safety of off-label extended dalbavancin treatment has also been reviewed by Cooper et. al [27]. In 144 patients who received at least two doses of dalbavancin and a median total dose of 3 g, one case of acute kidney injury was reported, but no other serious adverse events.

In the present case, there was a permanent loss of high frequency hearing. The exact pathogenesis of this hearing loss and its association with the dalbavancin treatment remain unclear, despite a temporal relationship. Certain drugs, such as aminoglycosides and cisplatin, are well known for their ototoxic effects due to their potential to cause death of hair cells. One important mechanism involves the generation of free radicals within the inner ear, resulting in damage to sensory cells [28]. This primarily affects the detection of high frequency sounds, which takes place at the basal turn of the cochlea. If extensive damage occurs, it can also affect the areas closer to the apex where low frequency sounds are detected [29].

To date, no cases of ototoxicity in relation to dalbavancin use have been reported [14, 15]. However, there may be an increased risk for patients receiving concomitant therapy with an ototoxic agent such as an aminoglycoside, There is, so far, only one report [13] of oto-vestibular symptoms associated with dalbavancin treatment; vertigo after long-term therapy that persisted after discontinuation of dalbavancin. Ototoxicity has otherwise not been reported during or after prolonged/long-term use of dalbavancin [24, 27, 30]. It should be noted that the ototoxicity also for long-term vancomycin treatment has been reported to be low in the absence of concomitantly administered ototoxic drugs, but there are reports on higher incidence of hearing loss with increasing age [20, 21, 31].

In this case report, the patient did not report any prior hearing problems; however, it was not possible to objectively confirm this due to the absence of a previous audiometry assessment, which represents a limitation. The underlying cause of his tinnitus remains unidentified. He had not previously been prescribed drugs known for their ototoxic potential, such as vancomycin and aminoglycosides. The patient initially had a baseline creatinine level ranging from 90 to 100 µmol/L, and an estimated glomerular filtration rate within the normal range. Surprisingly, on the day the first dose of dalbavancin was administered, the creatinine level has inexplicably increased to 119 µmol/L. However, one week later, the creatinine had returned to baseline level, 94 µmol/L. The unexplicable temporary decline in renal function may have played a role in the suspected dalbavancin toxicity observed in this patient.

PJIs require long-term antibiotic therapy, and dalbavancin is an attractive alternative to daily antibiotic intravenous administration for outpatient treatment, when no oral alternatives are available due to multidrug resistant pathogens, or if there is a risk of drug interactions. The downside to the very long half-life of dalbavancin is the risk of drug accumulation and/or persistence of high concentrations for an extended timeframe in the rare event of toxicity. We recommend assessment of each patient’s audiological history, and considering baseleine audiometry in selected cases, before initiating long-term therapy with dalbavancin. Audiometric assessments should also be repeated if the patient reports hearing detoriation during or after treatment. While we are not aware of any studies adressing the timeline of ototoxicity, ongoing research is being conducted on otoprotection in conjunction with the use of other ototoxic drugs such as cisplatin and aminoglycosides [29]. If available, therapeutic drug monitoring is highly recommended in clinical practice in order to optimize individual dosage regimens and avoid overdosing. Until optimal dosing intervals in long-term dalbavancin treatment are established, careful monitoring for signs of ototoxicity is recommended.

## Conclusions

We report a case of ototoxicity associated with dalbavancin treatment in a patient with a previous history of tinnitus. Dalbavancin is generally well tolerated, but attentiveness towards signs of ototoxicity is suggested, especially in off-label long-term prescription. Concomittant treatment with potentially ototoxic drus should be avoided. Audiometry may be considered in selected cases prior to long-term therapy with dalbavancin, in order to measure baseline hearing.

## Data Availability

All the data that support the findings of the case report have been provided in the article, and are also available from the corresponding author upon reasonable request.

## Abbreviations

CoNS: Coagulase-negative staphylococci
PJI: Prosthetic joint infection
DAIR: Debridement, antibiotics, and implant retention
PTA: Pure tone average
